# The relationship between general practitioner movement behaviours with burnout and fatigue

**DOI:** 10.1186/s12875-024-02289-5

**Published:** 2024-02-16

**Authors:** Richard S. Mayne, Gregory J. H. Biddle, Charlotte L Edwardson, Nigel D. Hart, Amanda J. Daley, Neil Heron

**Affiliations:** 1 School of Medicine, Dentistry and Biomedical Sciences, Queen’s University Belfast, Belfast, UK; 2 School of Sport, Exercise and Health Sciences, The Centre for Lifestyle Medicine and Behaviour, Loughborough University, Loughborough, UK; 3 Diabetes Research Centre, College of Life Sciences, University of Leicester, Leicester, UK; 4 Leicester Diabetes Centre, University Hospitals of Leicester NHS Trust, Leicester General Hospital, Leicester, UK; 5 School of Medicine, Keele University, David Weatherall Building, Keele, UK

**Keywords:** General practitioners, Sedentary behaviour, Physical activity, Burnout, Fatigue, activPAL

## Abstract

**Background:**

Physical inactivity is associated with feelings of burnout and fatigue, which in turn are associated with reduced performance among healthcare practitioners. This study explored movement behaviours of general practitioners (GPs) and the association between these behaviours with burnout and fatigue.

**Methods:**

GPs in Northern Ireland were asked to wear a thigh-worn accelerometer for seven days and complete validated questionnaires to assess the association between daily number of steps, time spent sitting and standing with feelings of burnout and fatigue.

**Results:**

Valid accelerometer data were obtained from 47 (77.0%) participants. Average workday sitting time, standing time and number of steps were 10.6 h (SD 1.5), 3.8 h (SD 1.3), and 7796 steps (SD 3116) respectively. Participants were less sedentary (8.0 h (SD 1.6)) and more active (4.7 h (SD 1.4) standing time and 12,408 steps (SD 4496)) on non-workdays. Fourteen (30.4%) participants reported burnout and sixteen (34.8%) reported severe fatigue. There were no significant associations between sitting, standing and step counts with burnout or fatigue (*p* > 0.05).

**Conclusion:**

GPs were less active on workdays compared to non-workdays and exhibited high levels of sitting. Feelings of burnout and fatigue were highly prevalent, however movement behaviours were not found to be associated with burnout and fatigue. Given the increased sedentariness among GPs on workdays compared to non-workdays, GPs should consider how they can improve their movement behaviours on workdays to help optimise their wellbeing.

**Supplementary Information:**

The online version contains supplementary material available at 10.1186/s12875-024-02289-5.

## Background

Working adults spend around 60% of their waking day sedentary, with those working in offices accumulating the most sedentary time [1]. General practitioners (GPs) have recently been identified as a sector of the workforce who spend a large proportion of their day sitting, for around 10.5 h per day on a workday [2]. GPs have high levels of sedentary time because of telephone and in-person consultations, as well as paper- and computer-based administrative tasks, all typically conducted while sitting [3]. GPs also have long core working hours each workday, reducing the opportunities for leisure-time physical activity (PA) [3].

High levels of sedentary behaviour (SB) (while awake in a sitting, lying or reclining posture in a state of low energy expenditure [4]), are associated with many physical and mental health problems [5–7] and increased all-cause mortality [7–13]. Many national and international guidelines therefore advise people to minimise SB, and break up prolonged periods of SB where possible [14]. Physical inactivity is associated with burnout and fatigue [15–23]. Burnout is a work-related phenomenon characterized by a severe loss of physical and mental energy [24]. Fatigue has been defined as an overwhelming sense of tiredness and exhaustion, with a lack of energy and associated impaired physical and/or mental functioning [25]. Burnout, fatigue and poor wellbeing among healthcare professionals are associated with reduced performance and patient-safety outcomes [26–30], yet burnout among GPs is consistently high across the globe [26, 27, 31–36]. Workload and job pressures are key contributors to burnout and fatigue [26, 27, 31–37], with primary care workload increasing in recent years due to a mismatch between capacity and demand [38, 39]. With primary care workload unlikely to reduce in the immediate future, it is important to consider other factors that can influence burnout and fatigue among GPs. Although lower levels of SB and higher levels of PA are associated with reduced burnout and fatigue in a range of occupations, including other healthcare professionals [15–21, 23], there is a lack of research among GPs. It could therefore be postulated that GPs with lower levels of SB and higher levels of PA may have less burnout and fatigue than GPs with higher levels of SB and lower levels of PA, due to the potential positive effect of PA, whereby an individual finds that increasing their PA helps them to reduce their feelings of burnout and fatigue.

The primary aim of this study was to explore the association between movement behaviours of GPs with their levels of burnout and fatigue, and to explore GPs perspectives on how working in primary care affects their health and wellbeing. This was achieved by describing accelerometer-assessed workday and non-workday movement behaviours and prevalence of burnout and fatigue among GPs.

## Methods

### Design

In accordance with the Strengthening the Reporting of Observational studies in Epidemiology (STROBE) guidance [40], a cross-sectional study was conducted. An online recruitment questionnaire was distributed using email and social media among GPs working in Northern Ireland during autumn 2020, as described in a previous paper (supplementary file 1) [2]. The 353 participants who completed the recruitment questionnaire (17.7% of GPs in Northern Ireland at the time of the study) were not given any reward and were not obligated to take part. Participants were asked to indicate whether they were willing to participate in an accelerometer study to gain detailed data regarding their SB and PA. Once recruitment was completed, the accelerometer and questionnaire study took place during spring 2021. The thigh-worn activPAL 3 micro (PAL Technologies, Glasgow, UK) accelerometer was used, which accurately measures sitting, standing and number of steps [41]. Participants were posted an accelerometer, adhesive waterproof dressings, and instructions detailing how to wear the accelerometer continuously, on the middle of the thigh, over a seven-day period while completing a contemporaneous sleep and work log. On completion, participants posted the accelerometer and sleep/work log back to the research team. While wearing the accelerometer, participants were also asked to complete an online questionnaire, developed for this study, regarding their physical and mental wellbeing (supplementary file 2). This included the single item burnout question [42], which has been validated among physicians as a measure of burnout against the Maslach Burnout Inventory [43, 44]. This allows participants to rate their level of burnout from one to five [42]. In this study, as with previous studies using this measure, scores of one and two represented no burnout and three to five represented burnout of increasing severity [42]. For example, a score of three represented the early stages of burnout (“I am definitely burning out and have one or more symptoms of burnout, such as physical and emotional exhaustion”), while a score of five represented later stages of burnout (“I feel completely burned out and often wonder if I can go on, I am at the point where I may need some changes or may need to seek some sort of help”) [42]. The questionnaire also included the 11-item Chalder Fatigue Scale [45, 46]. This is a short questionnaire which measures both physical and psychological fatigue [45, 46]. The questions ask about sensations and functionality, such as ‘Do you have problems starting things?’ and ‘Do you have difficulty concentrating?’ [45]. Each of the questions are answered on a 4-point scale ranging from no symptoms to maximum symptomology [45]. For the purposes of this study, a global binary fatigue score of four or more represented severe fatigue for the analysis, with a score of three or less representing the absence of severe fatigue [45, 46]. Furthermore, participants were asked open questions exploring how working in general practice affected their health and wellbeing.

### Inclusion and exclusion criteria

Inclusion criteria were as follows: employment as a GP partner, salaried GP, sessional or locum GP, or GPST (GP specialty trainee) working in general practice in Northern Ireland; having completed the online recruitment questionnaire; and having consented to being approached for a subsequent accelerometer study. Exclusion criteria were as follows: having a comorbidity that the participant felt would affect sedentary time; being on annual leave during the study; having participated in a previous accelerometer study, as described previously [2]; and being involved in contact sports that could damage the accelerometer.

### Analysis

Data from the activPAL were processed using Processing PAL V1.32 (University of Leicester, Leicester, UK, https://github.com/UOL-COLS/ProcessingPAL), which is a freely available java application that uses a validated algorithm to identify valid waking wear time and produce summary data for movement behaviours [47]. Average time spent sitting and standing per day, time spent sitting in uninterrupted sitting bouts lasting for over 30 min, and number of steps per day were exported. As required for previous accelerometer studies, a valid day required a minimum of 600 min of valid wear-time whilst awake [48]. For inclusion in the analysis, participants had to have a minimum of one valid workday (in which they worked at least one clinical session), and one valid non-workday during the time that they were wearing the accelerometer. This meant that both “full-time” and “part-time” GPs could be included in the study. Sleep/work logs were used in the analysis to determine workdays versus non-workdays and sleep and wake times. Questionnaire data were reviewed to ensure there were no duplicates, with IBM SPSS Statistics (version 29.0) used for statistical analyses. Baseline characteristics were described using mean (SD) for numerical data and counts (%) for categorical data. The distribution of numerical data was assessed visually using histograms and QQ plots. Dependent t-tests were used to analyse differences in movement behaviours between workdays and non-workdays. Multiple linear regression (for burnout) and binary logistic regression (for severe fatigue), analyses were undertaken to explore the relationship between total average daily sitting time, average daily time spent in prolonged sitting bouts lasting over 30 min, standing time, and step-counts (dependent variables) with burnout and fatigue (independent variables). Analyses were adjusted for age, sex, BMI and active workstation use. Free-text answers to all three open questions were categorised, based on the agreement of two authors (RSM and NH), into positive (e.g. participants felt happy with their overall levels of physical activity and sedentary behaviour), neutral (e.g. participants felt indifferent about the amount of time they spend sitting down in work), or negative (e.g. participants reported that working in general practice had detrimental effects on their health and wellbeing) responses.

## Results

### Recruitment

At the end of the initial recruitment questionnaire, 195 participants (55.2%) had indicated they were willing to be invited to take part in a subsequent accelerometer study to gain detailed data regarding their movement behaviours. Of these, 160 were approached by email for this study, as 35 had previously been approached for a different accelerometer study and had either participated in it or been ineligible. Seventy-five (46.9%) participants replied to the recruitment email for this accelerometer study. Eight did not meet the inclusion criteria: five were on maternity leave; two were on annual leave; and one was not currently working in general practice. Two stated they could not participate due to excessive workload, and one did not give a reason for declining participation when contacted. Three initially expressed an interest in participating, but later withdrew before the study commenced without providing a reason. Sixty-one participants from the 160 initially approached (38.1%) met the inclusion criteria for the study and were consented.

### Data capture and analysis

Of the 61 participants posted an accelerometer, valid accelerometer data were obtained from 47 (77.0%) participants. Data from 14 participants could not be analysed due to lost or malfunctioning accelerometers. Of the participants with valid accelerometer data, 46 (97.9%) completed the questionnaire.

### Sample characteristics

Summary characteristics of participants for whom valid accelerometer data were obtained are included in Table 1. Four participants (8.7%) used an active workstation, such as a sit-stand desk. Average age of participants was 41.4 years (Standard Deviation (SD) 8.3), average BMI was 25.0 kg/m² (SD 4.5). The characteristics of males and females were generally balanced. On workdays there was significantly higher average sitting time (10.6 h (SD 1.5) versus 8.0 h (SD 1.6), mean difference (MD) 2.6 h, 95% confidence interval (CI) 2.1–3.1; *p* < 0.001)), and time spent sitting in uninterrupted sitting bouts lasting for over 30 min (5.2 (SD 1.6) hours versus 4.0 (SD 1.7) hours, MD 1.2 h, 95% CI 0.7–1.7; *p* < 0.001), compared to non-workdays. Average standing time and step counts were significantly lower on workdays (3.8 h (SD 1.3) and 7795.5 steps (SD 3116.4)) compared to non-workdays (4.7 h (SD 1.4) and 12408.1 steps (SD 4496.7), MD 0.9 h (95% CI 0.4–1.3; *p* < 0.001) and 4612.6 steps (95% CI 3387.6-5837.5; *P* < 0.001). Burnout and fatigue were reported by 14 (30.4%) and 16 (34.8%) of the participants, respectively, for whom valid accelerometer data were available. The distribution of burnout scores was as follows: 32 participants had burnout scores of one or two, indicating no burnout; seven participants had a score of three, indicating moderate burnout; four participants had a score of four, indicating moderately severe burnout; three participants scored five, indicating severe burnout.

**Table 1:** Participant characteristics and baseline data

Characteristic	All participants
Sex, female/male, n (%)	24 (51.1) / 23 (49.9)
Age, years, mean (SD)	41.4 (SD 8.3)
BMI, kg/m² (SD)	25.0 (4.5)
Alcohol, units per week (SD)	6.8 (7.0)
Use of active workstation, no/yes, n (%)	42 (91.3) / 4 (8.7)
Average workday sitting time, hours (SD)	10.6 (1.5)
Average non-workday sitting time, hours (SD)	8.0 (1.6)
Average workday standing time, hours (SD)	3.8 (1.3)
Average non-workday standing time, hours (SD)	4.7 (1.4)
Average workday time spent sitting in prolonged uninterrupted bouts (> 30 min), hours (SD)	5.2 (1.6)
Average non-workday time spent sitting in prolonged uninterrupted bouts (> 30 min), hours (SD)	4.0 (1.7)
Average workday step-count (SD)	7795.5 (3116.4)
Average non-workday step-count (SD)	12408.1(4496.7)

SD = standard deviation, MD = mean difference

### Association between movement behaviours and burnout and fatigue

The relationship between overall sitting time, sitting time in prolonged bouts lasting over 30 min, standing time and step-counts with burnout and fatigue, accounting for age, sex, active workstation use and BMI, are shown in Tables 2 and 3. There were no associations between any activPAL assessed variables with burnout or fatigue (*p* > 0.05).

**Table 2:** Multiple linear regression exploring the relationship between sitting time, standing time and step-counts with burnout*

	β	SE	p
Workday sitting time (minutes)			
Burnout (per unit increase)	5.004	10.197	0.627
Workday sitting time in prolonged bouts (minutes)			
Burnout (per unit increase)	14.822	12.849	0.256
Non-workday sitting time (minutes)			
Burnout (per unit increase)	9.850	14.928	0.514
Non-workday sitting time in prolonged bouts (minutes)			
Burnout (per unit increase)	12.518	15.840	0.435
Workday standing time (minutes)			
Burnout (per unit increase)	7.238	9.193	0.436
Non-workday standing time (minutes)			
Burnout (per unit increase)	-0.180	13.663	0.990
Workday step-count (steps)			
Burnout (per unit increase)	-329.835	245.836	0.188
Non-workday step-count (steps)			
Burnout (per unit increase)	-461.549	356.021	0.203

* Model is adjusted for age, sex, BMI and workstation use

**Table 3:** Binary logistic regression exploring relationship between sitting time, standing time and step-counts with severe fatigue

	β	SE	Wald	p	OR (95% CI)
Workday sitting time	-0.015	0.013	1.188	0.621	0.986 (0.960–1.012)
Workday sitting time in prolonged bouts	0.008	0.008	0.842	0.359	1.008 (0.991–1.024)
Non-workday sitting time	-0.006	0.013	0.245	0.621	0.994 (0.969–1.019)
Non-workday sitting time in prolonged bouts	-0.003	0.010	0.126	0.722	0.997 (0.978–1.016)
Workday standing time	-0.016	0.012	1.740	0.187	0.984 (0.962–1.008)
Non-workday standing time	0.009	0.009	0.939	0.187	0.984 (0.962–1.008)
Workday step-count	0.000	0.000	2.929	0.087	1.000 (0.999-1.000)
Non-workday step-count	0.000	0.000	1.655	0.198	1.000 (1.000–1.000)

### Free-text responses

In response to the question “How do you feel about your overall levels of physical activity and sedentary behaviour?”, 23 (50%) responses were negative, 10 (21.7%) were neutral, and 13 (28.3%) were positive. An example of a negative response was “I am ashamed by how sedentary my life is. After a day working as a GP sitting down all day, I have very little energy or mental power to do anything else. I have a gym membership which I keep paying for but haven’t used in over 2 years,” (participant BL010). An example of a neutral response was “Outside work - lots of physical activity, inside - very sedentary,” (participant BL019). An example of a positive response was “Due to the pandemic I started being more active as a way of dealing with stress. The sedentary nature of our work has been on my mind for a while and the pandemic gave me the impetus to start moving,” (participant BL018).

When asked “How do you feel about the amount of time you spend sitting down in work?”, 42 (91.3%) responses were negative, 2 (4.3%) were neutral, and 2 (4.3%) were positive. The two positive responses were from participants with active workstations. An example of a negative response was “I hate the amount of time I sit at my desk and some days can easily sit there the entire day,” (participant BL016). An example of a neutral response was, “It’s not bad, as I tend to take walks often, but I’d prefer to move more often,” (participant BL009). An example of a positive response was, “I have significantly reduced this since I got my standing desk,” (participant BL058).

In response to the question “How does working in general practice affect your health and wellbeing?”, 32 (69.6%) responses were negative, 11 (23.9%) were neutral and 3 (6.5%) were positive. An example of a negative response was, “It’s not healthy- long hours sitting without breaks, constant pressure to get through the work and do so safely. Never finishing work at the end of the day and having admin work constantly hanging over and playing on my mind. I manage, but these things make it unhealthy,” (participant BL005). An example of a neutral response was, “Sometimes I feel tired and stressed but I also have a fulfilling career where I feel I can make a difference,” (participant BL057). An example of a positive response was, “I don’t feel it makes a big difference compared to other specialities, although working part time with weekends free allow me to pursue hobbies and time with family. I enjoy work so it is generally a positive experience,” (participant BL011).

## Discussion

This study explored the association between movement behaviours of GPs with their levels of burnout and fatigue, as well as GPs perspectives on how working in primary care affects their health and wellbeing. There were high levels of burnout and severe fatigue, however there were no statistically significant relationships between burnout and fatigue with GP movement behaviours. Qualitative responses revealed that most GPs had negative opinions about their own movement behaviours and how working in general practice affected their health and wellbeing.

GPs have high levels of workday sitting time, and significantly lower amounts of non-workday sitting time. A large proportion of workday sitting time is spent in uninterrupted sitting bouts lasting over 30 min. Average workday sitting time of 10.6 h was similar to desk-based workers in telecommunication, education and service industries and much higher than the recommended 24-hour movement guidelines for adults, which advise limiting sedentary time to eight hours or less per day [49–51]. However, average non-workday sitting time of 8.0 h per day was in line with these recommendations [49, 50]. Sitting time among participants in this study was consistent with a previous study exploring sedentary behaviour and physical activity among GPs [2]. The high amount of time (5.2 h, 49.1% of total daily sitting time) that participants spent sitting in prolonged bouts (> 30 min) on workdays is concerning given that prolonged, uninterrupted sitting can increase fatigue [52], reduce cognitive function [53] and worsen cardiometabolic markers [54]. Step counts were higher by an average of 4612.6 steps per day on non-workdays compared to workdays (7795.5 steps), demonstrating that GPs are more active on non-workdays compared to workdays and achieve non-workday step-counts above the 8,000 steps daily target for healthy adults [55]. This was supported by free text responses where participants reported purposefully undertaking more PA on non-workdays, due to feeling unsatisfied with their high levels of SB and low levels of PA on workdays.

Almost one third of participants reported burnout. This is within the upper range of the 6–33% prevalence estimate of burnout among GPs in a recent systematic review and meta-analysis of 22,177 GPs across 29 countries [33]. The reason for the high level of burnout is likely related to pressures of the job, with many free-text responses outlining high workload, long hours, and physical and mental health concerns related to working in general practice. Given that there was no statistically significant relationship between burnout and movement behaviours, a larger study would be needed to fully explore a hypothesis suggesting that by reducing SB and increasing PA, GPs could help reduce their feelings of burnout. An alternative hypothesis may be that GPs with higher workload have less opportunity for PA, with the underlying increased workload being the main reason for their higher levels of burnout.

There was a high prevalence of severe fatigue, affecting 34.8% of participants. This is similar to previous studies examining fatigue among nurses and prehospital emergency care providers [56, 57]. There is a lack of previous research examining fatigue among GPs, likely because fatigue has a more general definition than burnout, which is more specifically work related. There was a high degree of overlap between severe fatigue and burnout, with 68.8% of participants reporting both severe fatigue and burnout. Previous research has shown that individuals with concurrent severe fatigue and burnout are more likely to have worse health and work related outcomes than individuals reporting only one of these conditions [58].

Free text responses showed that most GPs felt negatively about their overall levels of SB and PA (50%), work-related SB (91.3%) and how working in general practice affected their health and wellbeing (69.6%). This is in keeping with previous research examining GPs’ opinions regarding SB and PA [3, 59] and is likely a contributing factor to their high levels of burnout and fatigue. However, it is important to note that many free-text responses described job related stresses and high workload as contributing to burnout and fatigue, which are difficult to address in the current climate of healthcare provision.

Strengths of this study include that demographic data of participants were similar to publicly available data regarding GPs in Northern Ireland [60] and accelerometry-measured movement behaviours are more accurate than self-reported measures [48]. This study has some limitations that need to be considered. The small sample size reduced the likelihood of finding a statistically significant association between GP movement behaviours and their levels of burnout and fatigue. The sample size was reduced due to the loss or malfunction of 14 accelerometers (23.0%), however this is fairly typical when using these research methods. Selection bias may have occurred, whereby participants were more interested in SB and PA than non-participants. Non-participants may not have had time to respond to the initial questionnaire due to excessive workload, which means they may have had higher levels of burnout and fatigue than participants.

## Conclusions

Despite there being widespread awareness of the harms of burnout and fatigue on health outcomes among clinicians and patients alike, levels of burnout and fatigue remain high among GPs. The reasons for this are multifactorial, with job related stresses and high workload likely key factors. As primary care workload is unlikely to reduce in the foreseeable future, it is important to consider other factors relating to burnout and fatigue. Given their high levels of sitting time on workdays, GPs could potentially consider how they could improve their movement behaviours on workdays to help optimise their overall wellbeing.

### Electronic supplementary material

Below is the link to the electronic supplementary material.

Supplementary Material 1

Supplementary Material 2

## Data Availability

The datasets used and analysed during the current study are available from the corresponding author following reasonable request.

## Abbreviations

GP: General practitioner
GPST: GP specialty trainee
SB: Sedentary behaviour
PA: Physical activity
SD: Standard deviation
MD: Mean difference
BMI: Body mass index
CI: Confidence intervals
