# Supplementary material for: The relationship between general practitioner movement behaviours with burnout and fatigue
Source: BMC Prim Care. 2024 Feb 16;25:60. doi: 10.1186/s12875-024-02289-5 (PMC10870505; doi:10.1186/s12875-024-02289-5)
Supplement: Supplementary file 1 — Supplementary Material 1 [file 12875_2024_2289_MOESM1_ESM.docx]

**Sedentary behaviour burnout and fatigue questionnaire**

1. **Please state your current height and weight. If you are not sure, please give an estimation.**

| Height in cm |  | Weight in kg |  |
| --- | --- | --- | --- |

1. **Do you smoke?**

**YES NO**

Cigarettes/day_______

1. **Do you drink alcohol?**

**YES NO**

Units/week___________

1. **Validated Burnout Question:** **single-item measure of burnout validated against the Maslach Burnout Inventory among physicians (Rohland et al.)**

Please select the statement that is most relevant to you:

- I enjoy my work. I have no symptoms of burnout.
- Occasionally I am under stress, and I don’t always have as much energy as I once did, but I don’t feel burned out.
- I am definitely burning out and have one or more symptoms of burnout, such as physical and emotional exhaustion.
- The symptoms of burnout that I’m experiencing won’t go away. I think about frustration at work a lot.
- I feel completely burned out and often wonder if I can go on. I am at the point where I may need some changes or may need to seek some sort of help.

1. **Validated Fatigue Questionnaire (****Chalder Fatigue Scale)**

We would like to know more about any problems you have had with feeling tired, weak or lacking in energy in the last month. Please answer ALL the questions by ticking the answer which applies to you most closely. If you have been feeling tired for a long while, then compare yourself to how you felt when you were last well. Please tick only one box per line.

|  | **Less than usual** | **No more than usual** | **More than usual** | **Much more than usual** |
| --- | --- | --- | --- | --- |
| Do you have problems with tiredness? |  |  |  |  |
| Do you need to rest more? |  |  |  |  |
| Do you feel sleepy or drowsy? |  |  |  |  |
| Do you have problems starting things? |  |  |  |  |
| Do you lack energy? |  |  |  |  |
| Do you have less strength in your muscles? |  |  |  |  |
| Do you feel weak? |  |  |  |  |
| Do you have difficulties concentrating? |  |  |  |  |
| Do you make slips of the tongue when speaking? |  |  |  |  |
| Do you find it more difficult to find the right word? |  |  |  |  |
|  | **Better than usual** | **No worse than usual** | **Worse than usual** | **Much worse than usual** |
| How is your memory? |  |  |  |  |

**Open (Free Text) Questions:**

1) How do you feel about your overall levels of physical activity and sedentary behaviour?

2) How do you feel about the amount of time you spend sitting down in work?

3) How does working in general practice affect your health and wellbeing?
