# Supplementary material for: The relationship between general practitioner movement behaviours with burnout and fatigue
Source: BMC Prim Care. 2024 Feb 16;25:60. doi: 10.1186/s12875-024-02289-5 (PMC10870505; doi:10.1186/s12875-024-02289-5)
Supplement: Supplementary file 2 — Supplementary Material 2 [file 12875_2024_2289_MOESM2_ESM.docx]

**Online Questionnaire**

*Section 1: Background Information*

1.Age

- Enter your answer

2.Gender

- Female
- Male
- Prefer not to say

3.Current job role

- GP
- GPST1
- GPST2
- GPST3

4.Current working environment(s)

- GP
- Paediatrics
- Emergency Medicine
- General Medicine
- Obstetrics and Gynaecology
- Psychiatry
- Other

5.Name of GP surgery where you are primarily based

Enter your answer

6.Number of clinical sessions per week in General Practice (on average)

- 0
- 1
- 2
- 3
- 4
- 5
- 6
- 7
- 8
- 9
- 10

7.Number of non-clinical sessions per week in General Practice (on average)

- 0
- 1
- 2
- 3
- 4
- 5
- 6
- 7
- 8
- 9
- 10

8.Number of sessions per week in roles outside of General Practice (on average)

- 0
- 1
- 2
- 3
- 4
- 5
- 6
- 7
- 8
- 9
- 10

9.Are you full time or less than full time?

- Full time
- Less than full time

10.What is your percentage of full-time equivalent?

Enter your answer

11.Current clinical working environment(s)

- General Practice
- Paediatrics
- Emergency Medicine
- General Medicine
- Obstetrics and Gynaecology
- Psychiatry
- Other

12.Name of GP surgery where you are primarily based

Enter your answer

*Section 2: Sedentary Behaviour Questions for GPs and GPSTs in General Practice*

The following questions are about activities you did over the past week while sitting, reclining or lying down. Do not count the time you spent sleeping or napping. Only count the time when this was your main activity. For example if you were watching television and surfing the internet, count it as television time or computer time, but not as both.

13.On a typical day when you worked in General Practice in the past week, how much time did you spend sitting or lying down and…

Watching TV or using a computer, tablet or smartphone? Count time watching videos, playing computer games, emailing or using the Internet inside and outside of work.

Please answer in hours and minutes. This includes the full day inside and outside of work.

Enter your answer

14.On a typical day when you worked in General Practice in the past week, how much time did you spend sitting or lying down and…

Reading a book or magazine? Only include reading during free time. Include reading done using electronic formats. Do not include time spent reading at work, during class time or while exercising.

Please answer in hours and minutes. This includes the full day inside and outside of work.

Enter your answer

15.On a typical day when you worked in General Practice in the past week, how much time did you spend sitting in a car, bus or train?

Please answer in hours and minutes. This includes the full day inside and outside of work.

Enter your answer

16.During the last 7 days, on a typical day when you worked in General Practice, how much OVERALL time did you usually spend sitting, reclining or lying down ? (Include time spent at work, at home, while doing course work, and during leisure time. This may include time spent sitting at a desk, visiting friends, reading, watching television).

Please answer in hours and minutes.

Enter your answer

17.On a typical DAY OFF WORK in the past week, how much time did you spend sitting or lying down and…

Watching TV or using a computer, tablet or smartphone? Count time watching videos, playing computer games, emailing or using the Internet.

Please answer in hours and minutes.

Enter your answer

18.On a typical DAY OFF WORK in the past week, how much time did you spend sitting or lying down and…

Reading a book or magazine? Include reading done using electronic formats. Do not include time spent reading during class time or while exercising.

Please answer in hours and minutes.

Enter your answer

19.On a typical DAY OFF WORK in the past week, how much time did you spend sitting in a car, bus or train?

Please answer in hours and minutes.

Enter your answer

20.During the last 7 days, on a typical DAY OFF WORK, how much OVERALL time did you usually spend sitting, reclining or lying down? Include time spent at home, while doing course work, and during leisure time. This may include time spent sitting at a desk, visiting friends, reading, watching television.

Please answer in hours and minutes.

Enter your answer

21.In work, would you prefer more or less time sitting down, or are you happy with things the way they are?

More time sitting

Same amount of time sitting as now

Less time sitting

22.In your General Practice working environment, do you have access to an "active workstation" such as a standing desk?

Yes

No

23.Would you ever consider using an "active workstation" (such as a standing desk) in work?

Yes

No

Maybe

24.When working as a GP in the current environment, do you feel that you now spend more time sitting, or less time sitting than prior to the COVID-19 pandemic?

More time sitting

Same amount of time sitting as before

Less time sitting

25.Why do you feel that you are spending more time sitting now than prior to the COVID-19 pandemic?

Enter your answer

26.Why do you feel that you are spending less time sitting now than prior to the COVID-19 pandemic?

Enter your answer

*Section 3: Sedentary Behaviour Questions for GPSTs Working in Secondary Care*

27.On a typical WORKING DAY in the past week, how much time did you spend sitting or lying down and…

Watching TV or using a computer, tablet or smartphone? Count time watching videos, playing computer games, emailing or using the Internet inside and outside of work.

Please answer in hours and minutes. This includes the full day inside and outside of work.

Enter your answer

28.On a typical WORKING DAY in the past week, how much time did you spend sitting or lying down and…

Reading a book or magazine? Only include reading during free time. Include reading done using electronic formats. Do not include time spent reading at work, during class time or while exercising.

Please answer in hours and minutes. This includes the full day inside and outside of work.

Enter your answer

29.On a typical WORKING DAY in the past week, how much time did you spend sitting in a car, bus or train?

Please answer in hours and minutes. This includes the full day inside and outside of work.

Enter your answer

30.During the last 7 days, on a typical WORKING DAY, how much OVERALL time did you usually spend sitting, reclining or lying down? (Include time spent at work, at home, while doing course work, and during leisure time. This may include time spent sitting at a desk, visiting friends, reading, watching television).

Please answer in hours and minutes.

Enter your answer

31.On a typical DAY OFF WORK in the past week, how much time did you spend sitting or lying down and…

Watching TV or using a computer, tablet or smartphone? Count time watching videos, playing computer games, emailing or using the Internet.

Please answer in hours and minutes.

Enter your answer

32.On a typical DAY OFF WORK in the past week, how much time did you spend sitting or lying down and…

Reading a book or magazine? Include reading done using electronic formats. Do not include time spent reading during class time or while exercising.

Please answer in hours and minutes.

Enter your answer

33.On a typical DAY OFF WORK in the past week, how much time did you spend sitting in a car, bus or train?

Please answer in hours and minutes.

Enter your answer

34.During the last 7 days, on a typical DAY OFF WORK, how much OVERALL time did you usually spend sitting, reclining or lying down? Include time spent at home, while doing course work, and during leisure time. This may include time spent sitting at a desk, visiting friends, reading, watching television.

Please answer in hours and minutes.

Enter your answer

35.In work, would you prefer more or less time sitting down, or are you happy with things the way they are?

More time sitting

Same amount of time sitting as now

Less time sitting

36.Thank you for taking the time to participate in this survey. Have you any other comments relating to this study?

Enter your answer
